# Quality of Life in Men With Congenital Adrenal Hyperplasia Due to 21-Hydroxylase Deficiency

**DOI:** 10.3389/fendo.2021.626646

**Published:** 2021-03-19

**Authors:** Myrthe J. M. Verhees, Manon Engels, Paul N. Span, Fred C. G. J. Sweep, Antonius E. van Herwaarden, Henrik Falhammar, Anna Nordenström, Emma A. Webb, Annette Richter-Unruh, Claire Bouvattier, Aude Brac de la Perrière, Wiebke Arlt, Nicole Reisch, Birgit Köhler, Marion Rapp, Nike M. M. L. Stikkelbroeck, Nel Roeleveld, Hedi L. Claahsen-van der Grinten

**Affiliations:** ^1^Department of Pediatrics, Amalia Children's Hospital, Radboud University Medical Center, Nijmegen, Netherlands; ^2^Department of Laboratory Medicine, Radboud Institute for Molecular Life Sciences (RIMLS), Radboud University Medical Center, Nijmegen, Netherlands; ^3^Radiotherapy and OncoImmunology Laboratory, Department of Radiation Oncology, Radboud Institute for Molecular Life Sciences (RIMLS), Radboud University Medical Center, Nijmegen, Netherlands; ^4^Department of Molecular Medicine and Surgery, Karolinska Institute, Stockholm, Sweden; ^5^Department of Endocrinology, Metabolism and Diabetes, Karolinska University Hospital, Stockholm, Sweden; ^6^Department of Women’s and Children’s Health, Division of Pediatric Endocrinology, Karolinska Institutet, Karolinska University Hospital, Stockholm, Sweden; ^7^Centre for Endocrinology, Diabetes and Metabolism, Birmingham Health Partners, Birmingham, United Kingdom; ^8^Institute of Metabolism and Systems Research (IMSR), University of Birmingham, Birmingham, United Kingdom; ^9^Sektion Kinderendokrinologie und Diabetologie, Klinik für Kinder- und Jugendmedizin der Ruhr-Universität Bochum im St. Josef-Hospital, Bochum, Germany; ^10^Endocrinologie Pédiatrique, Centre de Référence des Maladies Rares du Développement Sexuel, Hôpital Bicêtre, Université Paris-Sud, Le Kremlin-Bicêtre, France; ^11^Fédération d’Endocrinologie, Centre de Référence des Maladies Rares du Développement Génital, Groupement Hospitalier Est, Hopital Louis Pradel, Bron, France; ^12^Medizinische Klinik IV, Klinikum der Universität München, München, Germany; ^13^Klinik für Pädiatrie m.S. Endokrinologie und Diabetologie, Charité – Universitätsmedizin Berlin, Corporate Member of Freie Universität Berlin, Humboldt-Universität zu Berlin, and Berlin Institute of Health, Berlin, Germany; ^14^Klinik fur Kinder- und Jugendmedizin, Universitat zu Lubeck, Lubeck, Germany; ^15^Department of Internal Medicine, Division of Endocrinology, Radboud University Medical Center, Nijmegen, Netherlands; ^16^Department for Health Evidence, Radboud Institute for Health Sciences, Radboud University Medical Center, Nijmegen, Netherlands

**Keywords:** CYP21A2, WHOQOL BREF, quality of life, congenital adrenal hyperplasia (CAH), 21 hydroxylase deficiency

## Abstract

Congenital adrenal hyperplasia (CAH) due to 21-hydroxylase deficiency (21OHD) is a disorder of adrenal steroid biosynthesis, leading to hypocortisolism, hypoaldosteronism, and hyperandrogenism. Impaired quality of life (QoL) has been demonstrated in women with CAH, but data on men with CAH are scarce. We hypothesized that disease severity and poor treatment control are inversely associated with QoL. In this study, 109 men (16-68 years) with 21OHD were included. The WHOQOL-BREF questionnaire was used to measure self-reported QoL domain scores on a 0-100 scale, where higher scores reflect better QoL. QoL domain scores were compared to published data on healthy and chronically ill reference populations from France, Germany, the Netherlands, and the United Kingdom. Differences in QoL scores among groups of disease severity and treatment control were tested within the study population. Overall, the men with CAH in this study appeared to rate their QoL as good. Median domain scores were 78.6 (IQR: 67.9-85.7) for physical health, 79.2 (IQR: 66.7-87.5) for psychological health, 75.0 (IQR: 58.3-83.3) for social relationships, and 81.3 (IQR: 71.9-90.6) for environment. In general, these scores were similar to WHOQOL-BREF domain scores in healthy references and higher compared to chronically ill reference populations. The domain scores did not differ among genotype groups, but patients with undertreatment or increased 17-hydroxyprogestrone concentrations scored higher on several QoL domains (p<0.05). Patients treated with dexamethasone or prednisone scored higher on the physical health, psychological health, and social relationships domains, but not on the environmental domain. In conclusion, QoL domain scores appeared to be comparable to healthy reference populations and higher compared to patients with a chronic illness. QoL was not influenced by genotype, but undertreatment and use of dexamethasone or prednisone were associated with higher QoL.

## Introduction

Congenital adrenal hyperplasia (CAH) is an inherited, chronic disorder of adrenal steroid biosynthesis. The most common cause is a mutation in the *CYP21A2* gene leading to 21-hydroxylase deficiency (21OHD), which results in impaired production of cortisol and increased production of adrenal androgens, leading to virilization of the external genitalia in 46,XX individuals (1). Aldosterone production is also impaired to a variable degree, depending on the severity of the enzyme deficiency (1, 2).

Treatment consists of glucocorticoid substitution and, if necessary, substitution of mineralocorticoids as well (1). By treatment with glucocorticoids, the negative feedback on the pituitary gland is restored, leading to a decrease in adrenal androgen production. Mostly, however, supraphysiological dosages of glucocorticoids are necessary to decrease adrenal androgen production sufficiently. Therefore, balancing medical treatment between under- and overtreatment is important to prevent long-term consequences of chronic androgen exposure and chronic supraphysiological glucocorticoid exposure. Common long-term complications due to insufficient adrenal androgen suppression and insufficient glucocorticoid supplementation are disturbed pubertal development, reduced final height, decreased reproductive function, including testicular adrenal rest tumor development, and adrenal crises. Long-term complications due to chronic supraphysiological glucocorticoid exposure include decreased bone mineral density, increased risk of obesity, and cardiovascular morbidity (3–5).

In patients with CAH, several factors may affect quality of life (QoL), such as the development of long-term complications, the use of medication, and poor treatment control (6–8). Impaired QoL has been reported in patients with CAH, mostly in women [reviewed by Reisch et al. (3)]. Data on QoL in men with CAH are scarce, although separate analysis of QoL in male and female patients is important since clinical presentation and complications vary greatly between the two sexes. Results are contradictory with some papers describing impaired QoL (6, 9, 10), and others equal (11) or better (12) QoL in men with CAH compared to a control population [recently reviewed by Daae et al. (13)].

The dsd-LIFE study provides an opportunity to fill this knowledge gap by studying a large European multicenter cohort of men with CAH in which we assessed QoL using the WHOQOL-BREF questionnaire. We hypothesized that QoL would be impaired in men with CAH compared to healthy control populations, and that genotype, medication regimen, and treatment control would be associated with QoL.

## Methods

### Patients

Adult men with CAH were included from the dsd-LIFE study, a cross-sectional clinical outcome study of individuals with disorders/differences in sex development (DSD). The methodological background of the dsd-LIFE study is described in more detail elsewhere (14). Fourteen study centers in six European countries (France (n=4), Germany (n=4), Poland (n=2), the Netherlands (n=2), Sweden (n=1), and the United Kingdom (UK) (n=1)) included participants with DSD (n=1040) from February 2014 until September 2015. In addition, male patients with CAH (karyotype 46,XY) were invited to participate in the dsd-LIFE study as they may face similar challenges as DSD patients, even though they are not classified as such. In total, 121 men with CAH (karyotype 46,XY), aged 16 - 68 years, were included. Written informed consent was obtained from all participants. The study was approved by the medical ethics committee at the Charité Universitätsmedizin Berlin (reference number EA2/069/13) and the local ethics committees of the other study centers as appropriate for each country.

Patients were investigated at their local medical center and treated according to the Endocrine Society guidelines (1). All patients underwent medical examination and filled out several questionnaires, including the WHOQOL-BREF (15). Additional data were retrieved from medical records. General patient characteristics and clinical parameters included: country of inclusion, height, weight, BMI, age, age at diagnosis, *CYP21A2* genotype, medication use, subjective treatment control, satisfaction with care in childhood, smoking behavior, work, leisure time activities, sports, and educational level. The variable age was dichotomized into <30 years and ≥30 as described in other studies (9, 11). The patients were classified into genotype groups null (0), A, B, and C, based on the residual enzymatic activity (16). Enzyme activity is lowest in genotype 0 (0% remaining activity), with increasing activity from A through C (17). Therefore, patients with genotype group 0 are most severely affected, while genotype groups A, B, and C have decreasing severity. The patients’ educational levels were established according to the EU classification as low, medium, and high (18).

### Reference Populations

As matched control groups were not available in the dsd-LIFE study, this study used control groups from the literature. QoL data on female patients with CAH were derived from an earlier DSD-LIFE study on QoL in patients with DSD (19). This study included a sample of 226 female patients with CAH from France, Germany, the Netherlands, Sweden and the UK. In addition, healthy as well as chronically ill populations from France (20), Germany (21), the Netherlands (22), and the UK (23) were used for comparison. Reference populations from the Netherlands and the UK contained both men and women, while France and Germany reported gender-specific QoL scores. For France, data of self-reported healthy (n=5167) and chronically ill (n=1638) adult men, of which 656 were young adults (18-24 years) and 897 were elderly people (65-75 years), were derived from the National Health Barometer 2005, a periodic study by the French National Institute for Preventive and Health Education (20). For Germany, data of 925 men from a representative urban sample of the adult general population were available, including 124 young adults (18 to 25 years) and 155 elderly people (≥66 years). Additionally, 261 men and women from this representative urban sample reporting a physical chronic disease were used as a chronically ill reference population (21). For the Netherlands, data from a healthy control group were used, matched for age and sex ratio (mean age 34.8 years) to a sample of patients with a mental chronic disease. The matched control group was taken from a pooled data set based on Dutch general population studies (1999–2002) (22). For the UK, a study including healthy and non-healthy people from all over the UK was available. The healthy people included students and student nurses. The age range for the entire cohort was 16–105 years with a mean age of 45 years; 64% of the study participants were women (23).

### WHOQOL-BREF

To assess QoL, the WHOQOL-BREF questionnaire was used according to guidelines provided by the World Health Organization (15). The WHOQOL-BREF is a shortened version of the WHOQOL-100, consisting of 24 questions concerning QoL on four different domains: physical health, psychological health, social relationships, and environment. Questions are rated on a 5-point Likert scale and domain scores represent the mean score of the items within each domain. The scores are multiplied by four, resulting in scores ranging from 4 to 20, to be directly comparable with scores derived from the WHOQOL-100. These scores can then be converted to a 0-100 scale, with high scores reflecting good QoL.

### Treatment Control

Treatment accuracy was estimated by the treating physicians in subjective scores: undertreatment, accurate treatment, or overtreatment. Blood hormone concentrations were also used to indicate treatment accuracy as high concentrations of androstenedione and 17-hydroxyprogesterone (17OHP) indicate inadequate adrenal androgen suppression. Therefore, blood samples were obtained at study inclusion. Samples were mostly taken in the morning, before intake of the glucocorticoid medication (14). Androstenedione and 17OHP concentrations were measured in the local hospital laboratory and compared to local reference values. The results were reported as “below reference range”, “within reference range”, “above reference range up to twice the upper limit”, and “more than twice the upper limit of the reference range”. To increase the number of patients per category, we combined the latter two categories into the category ‘above reference range’.

### Statistical Analysis

SPSS Statistics 25 (SPSS Inc., Chicago, IL, USA) was used for all analyses. First, descriptive analyses were performed for each variable. After checking missing data and the distributions of the continuous background variables and QoL scores for normality, median and interquartile ranges (IQR = Q1-Q3) were calculated. The overall and country-specific median QoL scores for all 4 domains were compared to the reference populations described above without further statistical analyses. Following, the QoL domain scores (physical, psychological, social relationships, and environment) were compared between male CAH patients with different ages, educational levels, and treatment regiments using the Mann-Whitney-U test. For BMI and glucocorticoid treatment dosages related to the QoL domain scores, Spearman correlation coefficients were calculated. Overall differences in QoL domain scores among CAH patients with different genotypes and various glucocorticoid treatment groups were assessed with the Kruskal-Wallis test or the Jonckheere-Terpstra test, and post-hoc Mann-Whitney-U tests were applied if the p-value was ≤0.15. In general, p-values <0.05 were considered statistically significant, but due to multiple testing the p-values from the latter analyses should be interpreted with caution.

## Results

### Quality of Life in Patients With Congenital Adrenal Hyperplasia

After exclusion of 10 men with CAH who did not complete the WHOQOL-BREF and two men with an 11ß-hydroxylase deficiency, 109 men with CAH due to 21-hydroxylase deficiency were included in this study on QoL. General characteristics are shown in **Table 1**.

**Table 1:** General characteristics of 109 men with CAH.

Variable	N		Result Median (IQR) or Number (%)
**Age** (years)	109		29.0 (21.0–40.8)
**Country of inclusion**	109	France Germany Netherlands Sweden United Kingdom	30 (27.5%) 46 (42.2%) 12 (11.0%) 9 (8.3%) 12 (11.0%)
**Height** (cm)	108		170.0 (166.3–175.0)
**BMI** (kg/m^2^)	108		25.4 (22.6–29.8)
**Educational level**	98	Low Medium High	14 (14.3%) 59 (60.2%) 25 (25.5%)
**Genotype**	109	Group 0 Group A Group B Group C No mutation analysis performed	22 (20.2%) 33 (30.3%) 30 (27.5%) 3 (2.8%) 21 (19.2%)
**Glucocorticoids**	109	No glucocorticoids Hydrocortisone Prednisolone Prednisone Dexamethasone More than 1 glucocorticoid*	5 (4.6%) 63 (57.8%) 15 (13.8%) 12 (11.0%) 7 (6.4%) 7 (6.4%)
**Glucocorticoid dose** (mg/day)**	104		27.9 (22.3–31.5)
**Mineralocorticoids**	109	Fludrocortisone No fludrocortisone	79 (72.5%) 30 (27.5%)
**Fludrocortisone dose** (mcg/day)	79		100.0 (75.0–150.0)

Continuous variables are displayed as median (IQR: Q1–Q3). Categorical variables are displayed as number of participants with percentage. Patients were classified according to severity of the disease into genotype groups null (0) through group C (17).

21OHD, 21-hydroxylase deficiency; BMI, body mass index; IQR, interquartile range.

*Six patients used hydrocortisone and dexamethasone, and one patient used hydrocortisone and prednisolone. **Hydrocortisone equivalent scores were calculated for the total dose of glucocorticoids used per day (24).

#### QoL Domain Scores in Patients With CAH

Overall, the men with CAH rated their QoL as good with median domain scores of 78.6 (IQR: 67.9–85.7), 79.2 (IQR: 66.7–87.5), 75.0 (IQR: 58.3–83.3), and 81.3 (IQR: 71.9–90.6) for physical health, psychological health, social relationships, and environment, respectively (**Table 2**). The country-specific median scores appeared to be lowest on all domains in patients from the UK and highest in Dutch patients (**Table 2**). Men <30 years scored higher (median score 75.0, IQR 60.4 – 91.7) on the social relationships domain compared to men ≥30 years (median score 66.7, IQR 50.0 – 83.3, p=0.03). For the domains physical health and psychological health, a few not statistically significant differences were seen between educational levels (**Supplement A**). No statistically significant correlation coefficients were found between the QoL domain scores and BMI and treatment dosages, except for the environmental domain and BMI (r=-0.26, p=0.006).

**Table 2:** QoL domain scores—men with CAH and country-specific healthy and chronically ill reference populations.

			Physical health	Psychological health	Social relationships	Environment
**Total cohort**	**Men with CAH**	**n = 109**	**78.6 (67.9–85.7)**	**79.2 (66.7–87.5)**	**75.0 (58.3–83.3)**	**81.3 (71.9–90.6)**
**DSD-LIFE** (19)	Women with CAH	n = 211	68.1 ± 18.9	65.6 ± 18.4	64.8 ± 20.5	74.1 ± 15.7
**France** (20)	**CAH**	**n = 30**	**71.4 (63.4–78.6)**	**77.1 (66.7–87.5)**	**66.7 (50.0–75.0)**	**76.6 (71.1–82.0)**
	Healthy	n = 5,157	81.6 ± 0.2^#^	69.5 ± 0.2^#^	75.6 ± 0.2^#^	-
	ill	n = 1,638	68.4 ± 0.4^#^	67.5 ± 0.3^#^	71.8 ± 0.4^#^	-
**Germany** (21)	**CAH**	**n = 46**	**83.9 (75.0–92.9)**	**79.2 (70.8–87.5)**	**75.0 (58.3–83.3)**	**84.4 (75.0–90.6)**
	Healthy*	n = 925	78.8 ± 16.9	75.9 ± 14.7	72.3 ± 18.2	71.2 ± 14.3
	ill^	n = 261	53.4 ± 20.3	62.7 ± 16.3	68.0 ± 16.9	67.2 ± 13.4
**Netherlands** (22)	**CAH**	**n = 12**	**89.3 (66.1–96.4)**	**81.3 (59.4–91.7)**	**87.5 (68.8–100.0)**	**89.1 (67.2–96.9)**
	Healthy^	n = 218	70.1 ± 11.9	64.8 ± 9.0	71.3 ± 13.4	74.0 ± 9.3
**Sweden**	**CAH**	**n = 9**	**82.1 (67.9–85.7)**	**79.2 (66.7–81.3)**	**66.7 (50.0–79.2)**	**81.3 68.8–93.8)**
**United Kingdom** (23)	**CAH**	**n = 12**	**58.9 (45.5–85.7)**	**60.4 (32.3–72.9)**	**41.7 (10.4–75.0)**	**65.6 (52.3–78.1)**
	Healthy^	n = 1,328	76.5 ± 16.2	67.8 ± 15.6	70.5 ± 20.7	68.2 ± 13.8
	ill^	n = 524	67.8 ± 19.6	67.7 ± 16.1	70.1 ± 19.7	71.1 ± 15.5

Median WHOQOL-BREF domain scores and interquartile range (Q1–Q3) for the overall cohort and from country-specific analysis were calculated. These scores were compared to mean domain scores plus standard deviation (SD) or standard error of the mean (SEM) of a cohort of female patients with CAH from the literature (19) and to country-specific reference populations from the literature (20–23). If available, both healthy and chronically ill reference populations were used. Chronically ill reference populations comprised patients with a chronic physical illness; in the United Kingdom, only patients with a chronic endocrine disorder were included. Bold, Subgroups of men with CAH.

^#^Standard error of the mean instead of standard deviation.

*The German healthy reference population contained all male patients from the cohort including chronically ill patients.

^Quality of Life scores were obtained from a reference population containing both men and women.

#### Quality of Life Domain Scores Among Different Genotypes

The QoL domain scores differed only slightly among the different genotype groups, and no statistically significant differences were observed (**Supplement B**). Interestingly, the median QoL domain scores seemed among the highest for all four domains in men with genotype 0, who are affected most severely.

#### Quality of Life Domain Scores for Medication Use and Treatment Control

**Figure 1:**
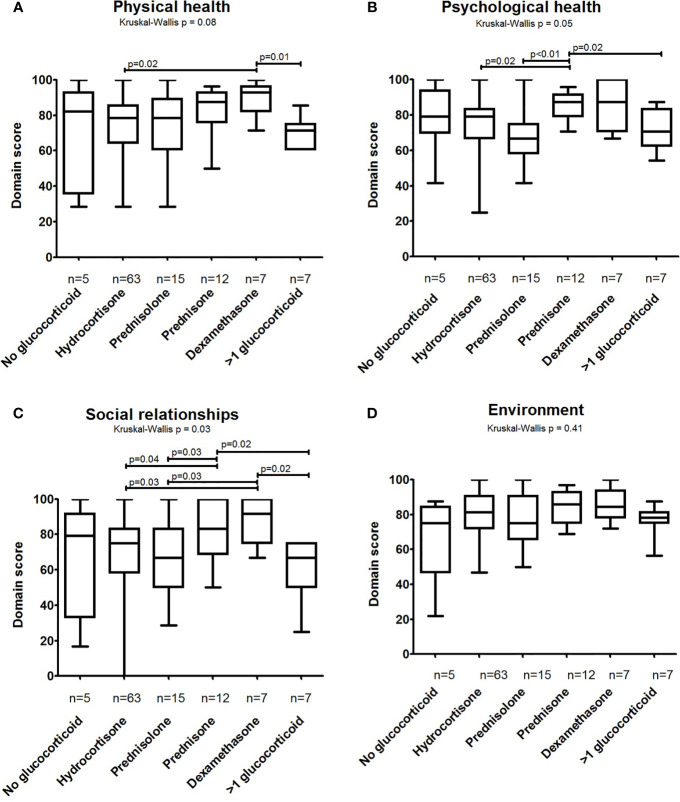
Quality of Life domain scores of men with CAH divided by glucocorticoid type. WHOQOL-BREF scores in different glucocorticoid type groups were calculated for four different domains: **(A)** physical health, **(B)** psychological health, **(C)** social relationships, and **(D)** environment. WHOQOL-BREF scores were converted to a 0–100 scale where higher scores reflect better QoL. Boxes represent median and 25th–75th percentiles, while whiskers show minimum–maximum domain scores. Differences among the groups were assessed using the Kruskal–Wallis test (p-value mentioned above the graph). When applicable, the Mann–Whitney-U test was used to provide some insight into the main differences between groups (p-values above bars; only p-values ≤ 0.05 are shown, but should be interpreted with caution due to the number of tests performed).

**Figures 1A-D** show the results for the QoL domain scores stratified by glucocorticoid treatment group. No statistically significant differences were seen in the environmental domain scores (Kruskal-Wallis p=0.41), but the p-values from the Kruskal-Wallis tests pointed towards differences among the treatment groups in the physical health (p=0.08), psychological health (p=0.05), and social relationships (p=0.03) domains. Patients treated with prednisone and dexamethasone had the highest scores in these domains. The largest differences were observed between dexamethasone and >1 glucocorticoid treatment in the physical health and social relationships domains and between prednisone and prednisolone treatment in the psychological health domain. Patients who were treated with fludrocortisone had lower QoL scores on the physical health domain (n=79, median: 78.6, IQR: 64.3-85.7) compared to patients who did not receive fludrocortisone (n=30, median: 82.1, IQR: 74.1-92.9; p=0.03). Subjective treatment accuracy did not seem to influence the QoL scores greatly (**Table 3**). However, undertreated patients had higher domain scores on the social relationships and environmental domains compared to patients who were accurately treated (p=0.04 and p=0.01, respectively). Patients with 17OHP concentrations above the reference range, indicating inadequate adrenal suppression, reported higher domain scores on the psychological health domain compared to patients with 17OHP concentrations within reference range (p=0.01) (**Table 3**).

**Table 3:** QoL domain scores—subgroups based on subjective and objective treatment accuracy in men with CAH.

	Subjective treatment accuracy			p-Value	17-OHP concentration		p-Value
	Undertreatment (n = 13)	Accurate treatment (n = 71)	Overtreatment (n = 7)		Within reference range (n = 23)	Above reference range (n = 57)	
**Physical health**	82.1 (67.9–98.2)	78.6 (67.9–85.7)	71.4 (53.6–92.9)	0.21	78.6 (64.3–85.7)	78.6 (71.4–91.1)	0.20
**Psychological health**	79.2 (72.9–85.4)	75.0 (66.7–87.5)	87.5 (70.8–91.7)	0.61	70.8 (58.3–79.2)	79.2 (70.8–87.5)	**0.01**
**Social relationships**	83.3 (70.8–100.0)	75.0 (58.3–83.3)	66.7 (58.3–83.3)	0.10	66.7 (50.0–75.0)	75.0 (58.3–83.3)	0.06
**Environment**	87.5 (79.7–95.3)	81.3 (71.9–87.5)	84.4 (71.9–93.8)	0.15	78.1 (68.8–81.3)	81.3 (75.0–90.6)	0.05

Median WHOQOL-BREF domain scores and interquartile range (Q1–Q3) for the subgroups based on subjective and objective treatment accuracy were calculated. 17-OHP, 17-hydroxyprogesterone. Subjective treatment accuracy groups were compared using the Jonckheere–Terpstra test for trend, while 17-OHP concentration subgroups were compared using the Mann–Whitney-U test. Bold: p < 0.05.

### Quality of Life in Patients With Congenital Adrenal Hyperplasia Compared With Reference Populations

#### QoL Domain Scores in Male Patients With CAH Compared to Female Patients With CAH

QoL domain scores appeared to be higher in male patients with CAH compared to female patients with CAH on all four domains (**Table 2**).

#### Quality of Life Domain Scores in Patients With Congenital Adrenal Hyperplasia Compared With Healthy Reference Populations

The physical health domain scores appeared to be similar in the total cohort of men with CAH compared to healthy subjects from France, Germany, and the UK. In country-specific scores, patients with CAH from France and the UK scored lower than their respective reference populations, whereas this was reverse for the Dutch CAH patients. (**Table 2**).

On the psychological health domain, the total group of men with CAH appeared to have a similar score as the healthy reference population from Germany, but a higher QoL score compared to healthy references from France, the Netherlands, and the UK (**Table 2**). Men with CAH from France and the Netherlands appeared to have rated their psychological health higher than the corresponding healthy reference populations, whereas men with CAH from the UK seemed to score lower than the corresponding healthy reference population.

For the social relationships domain, the score of the cohort of men with CAH appeared to be similar to the scores of healthy subjects from all reference populations. The country-specific scores were much higher and lower for CAH patients than for healthy references in the Netherlands and the UK, respectively.

On the environmental domain, the total cohort of men with CAH appeared to report higher scores compared to healthy reference populations from Germany, the Netherlands, and the UK, with CAH patients from Germany and the Netherlands rating their QoL much higher compared to the corresponding healthy reference populations. Men with CAH from the UK seemed to have similar scores as the healthy UK reference population.

#### QoL Domain Scores in Patients With CAH Compared to Chronically Ill Reference Populations

In comparison to chronically ill reference populations from France, Germany, and the UK (the latter comprising patients with diabetes only), higher scores were reported in the total cohort of men with CAH and in the country-specific cohorts for France, Germany, the Netherlands, and Sweden in all but one domain. In the social relationships domain, CAH patients from France, Sweden, and the UK seemed to score lower than most chronically ill reference populations. Men with CAH from the UK appeared to have lower median scores compared to the corresponding chronically ill reference population in all domains.

## Discussion

This is the first international multicenter study examining QoL using the WHOQOL-BREF questionnaire in a large cohort of adult male patients with CAH. This study shows that men with CAH rate their QoL as good. The overall scores appeared to be similar to scores obtained with the WHOQOL-BREF questionnaire in healthy reference populations from France (20), Germany (21), and the UK (23), and higher compared to a healthy reference population from the Netherlands (22). We also presented data on chronically ill reference populations, as having a chronic disease may affect expectations of life, leading to higher QoL scores due to overrating (25). The data showed that men with CAH in general appeared to rate their QoL higher compared to female patients with CAH and patients with other chronic diseases.

Although men with CAH may face different complications of their chronic disease and often require lifelong therapy, they do not seem to report a worse QoL on the physical and psychological health domains compared to healthy or chronically ill references. CAH patients from the UK form an exception, which is in line with an earlier study of Arlt et al. who found that only a minority of CAH patients in the UK receive optimal specialist endocrine care (6). The findings suggest that psychological wellbeing does not seem to be largely affected in men with CAH, which is in contrast to previously reported higher prevalence rates of psychiatric morbidity in men with CAH (26). Patients with mental health issues, however, might be less likely to participate in studies or fill out questionnaires on QoL, and may be underrepresented in the dsd-LIFE database. Social relationships as well as environmental QoL domain scores appeared to be similar or higher in men with CAH compared to reference scores, but several country-specific scores for social relationships were lower. In contrast, higher QoL domain scores were observed in patients with CAH < 30 years old compared to patients ≥ 30 years on the social relationships domain. This is supported by Skevington et al., who report decreasing WHOQOL-BREF domain scores with increasing age in a large healthy international cohort (27).

In country-specific comparisons, we observed high scores on all four domains in the Dutch men with CAH compared to men with CAH from other countries, along with scores of German and Swedish patients on some domains. These results are not reflected in the reference literature, as the Dutch healthy reference population had the lowest scores on the physical and psychological health domains compared to reference populations from the other countries and similar scores on the other domains (20–23). Possibly, the Dutch reference study does not accurately reflect the current Dutch general population, as the data were collected 15 years earlier than the dsd-LIFE study from a small sample. Another notable finding was that UK patients with CAH reported low scores on all four domains. Compared to healthy references from the UK (23), men with CAH rated QoL rather comparable on the environmental domain, but the scores on the other domains seemed much lower. However, the healthy reference population from the UK consisted of both male and female university students and student nurses, most likely resulting in a young and highly educated cohort. This may have led to overestimation of the QoL scores for the UK general population.

A few other studies reported QoL in men with CAH, but these used different questionnaires (13). Our results indicate a good QoL in men with CAH, which corresponds to the results found by Falhammar et al. (11), although impaired QoL has also been reported (6, 9, 10). Strikingly, Reisch et al. showed impaired QoL in 36 men with CAH on the GBB-24, whereas QoL measured with the HADS and SF-36 did not differ from a healthy reference population (10). This stresses the importance of using similar questionnaires to assess QoL in patients with CAH to make comparison among different study populations possible.

The QoL in men with CAH in our study was also higher compared to patients with primary adrenal insufficiency, although QoL in the latter study was not measured by WHOQOL-BREF (10). Furthermore, QoL measured by WHOQOL-BREF in men with CAH was higher compared to patients with DSD, including Turner Syndrome, Klinefelter syndrome, XY female DSD, and XY male DSD, as described in another study of dsd-LIFE (19). One of the differences between these diseases is the presence of increased adrenal steroid precursors in CAH, in contrast to patients with primary adrenal insufficiency or other forms of DSD. Previously, we showed that several adrenal steroid precursors that are elevated especially in CAH patients with poor hormonal control are able to activate the glucocorticoid receptor, which might explain why patients with CAH experience fewer complications of their cortisol deficiency than expected (28), possibly leading to better QoL. Furthermore, most males with CAH do not report complaints from testosterone deficiency as they have sufficient androgens from adrenal origin. Our observations argue in favor of the common treatment strategy of a more individualized treatment approach. In patients with CAH who want to achieve good fertility, adrenal androgen levels should be within the normal reference range, even when supraphysiological dosages of glucocorticoids are necessary. In older patients, in whom fertility issues are no longer relevant, more physiological dosages of glucocorticoids could be used to prevent long-term complications of glucocorticoid treatment.

The QoL observed in men with CAH in this study was also higher compared to the QoL observed in women with CAH, as described in another study of dsd-LIFE (19). This may reflect the differences in clinical presentation and complications, as female patients have more problems due to increased adrenal androgens, such as virilization and masculinization, which consequently require corrective surgery and may affect QoL negatively.

The QoL domain scores did not differ among the different genotypes, confirming the findings in a previous study (11). However, we did find that fludrocortisone therapy, given to the most severely affected CAH patients, was associated with lower QoL scores on the physical domain. Remarkably, men with genotype 0, who are most severely affected, had relatively high median QoL scores (**Supplement B**). Possibly, altered expectations of life are more pronounced in this group of patients, who received their diagnosis directly postnatally. Alternatively, one may speculate that an early start of follow-up may have led to improved QoL, which is in line with studies showing that a late diagnosis impairs QoL in male CAH patients (11) and that neonatal screening for CAH improves fertility in men with CAH (29).

Patients on dexamethasone or prednisone seemed to rate their QoL on the physical health, psychological health, and social relationships domains higher compared to patients that used other types of glucocorticoids, but no differences were observed on the environmental domain. This is in accordance with the finding of Falhammar et al. (11). In contrast, Han et al. reported lower QoL in patients using dexamethasone (30). However, the latter patients could have been on any regimen including dexamethasone mono-therapy as well as multiple glucocorticoids, which may have influenced the QoL scores negatively. In addition, our dexamethasone group contained only seven men, which might have skewed the results positively. Men with CAH using multiple glucocorticoids scored among the lowest on the physical health and social relationships domains. Possibly, patients with poor hormonal control are more likely to be treated with more than one glucocorticoid eventually. We observed higher scores among patients who were undertreated according to the subjective rating of the treating physician, as well as among patients with increased 17OHP concentrations compared to patients with normal 17OHP concentrations, but we did not find an association with androstenedione concentrations. Falhammar et al. also reported better QoL (PGWB questionnaire) in men with CAH in undertreated compared to overtreated patients (11). We hypothesize that male patients with CAH suffer less from androgen excess due to undertreatment than from glucocorticoid excess in overtreatment. Furthermore, elevated precursor steroids, such as 17OHP which also have glucocorticoid activity, may partially compensate glucocorticoid deficiency in this patient group (31). However, this potential association between treatment control and QoL should be re-evaluated in future studies, as the subjective rating of treatment control used in this cohort contains heterogeneity among treating physicians, normal 17OHP levels do not reflect accurate treatment, and an association with androstenedione concentrations was absent.

Although we were able to include a large cohort of adult male patients with CAH, our study design was mainly based on descriptive analyses and did not include a reference population. Therefore, we compared our domain scores with scores reported in the literature, although only mean domain scores were available and the German and France cohorts only reported gender-specific QoL scores. This complicated the comparisons with our median domain scores as no statistical analyses were possible. In addition, no data on educational level of the reference populations were available. Although all male patients with CAH were invited to participate in some centers, it is likely that some declined because they do not identify with DSD patient characteristics. Furthermore, all centers involved in the dsd-LIFE study are tertiary care centers. Both of these factors may have led to selection of the patient group towards including more severely affected patients. However, this substantiates our findings of good QoL in patients with CAH even more. In contrast, selection of highly motivated patients may have occurred, leading to overestimation of QoL. Until now, no cut-off values for ‘good QoL’ have been described in the literature for the WHOQOL-BREF.

## Conclusions

In conclusion, adult male patients with CAH, who were treated according to the international guidelines, rated their QoL as good in this study. Most of their QoL domain scores appeared to be comparable to healthy reference populations and higher compared to female patients with CAH and patients with other chronic illnesses. QoL was not influenced by genotype, but undertreatment and use of dexamethasone or prednisone were associated with higher QoL. Further studies are necessary to investigate factors that may influence QoL in CAH patients in more detail.

## Author’s Note

We publish this paper in memoriam of and with the greatest thanks to PD Dr. Birgit Köhler (Charité Universitätsmedizin, Berlin), the principle investigator of the European consortium dsd-LIFE and the initiator and co-author of this paper, who died in March 2019 from severe illness. We honour Birgit Köhler’s dedicated leadership and the energy and enthusiasm she put into the dsd-LIFE project and into the promotion of collaboration of clinicians, patients, and support groups—aiming to improve clinical care for “differences/disorders of sex development.” The authors are deeply sorrowed about this loss and state their gratefulness to the outstanding work of Birgit Köhler.

## Data Availability Statement

The original contributions presented in the study are included in the article/**Supplementary Material**. Further inquiries can be directed to the corresponding author.

## Collaborative Authors

Members of dsd-LIFE group are: Birgit Kohler, Berlin; Peggy Cohen-Kettenis and Annelou de Vries, Amsterdam; Wiebke Arlt, Birmingham; Claudia Wiesemann, Gottingen; Jolanta Slowikowska-Hilczer, Lodz; Aude Brac de la Perriere, Lyon; Charles Sultan and Francoise Paris, Montpellier; Claire Bouvattier, Paris; Ute Thyen, Lubeck; Nicole Reisch, Munich; Annette Richter-Unruh, Munster; Hedi Claahsen-van der Grinten, Nijmegen; Anna Nordenstrom, Stockholm; Catherine Pienkowski, Toulouse; and Maria Szarras-Czapnik, Warsaw.

## Author Contributions

MV, ME, and HC-G were involved in the conception and design of the study. All authors were involved in collecting the data. ME and MV performed the data analysis, interpreted the data, and drafted the manuscript. NR contributed to the statistical analysis and interpretation of the data. MV, ME, HC-G, PS, FS, AH, NRo, NS, HF, and MR critically revised the manuscript at different stages in the writing process. All authors contributed to the article and approved the submitted version.

## Funding

This work was funded by the European Union Seventh Framework Programme (FP7/2007-2013) under grant agreement n° 305373.

## Conflict of Interest

The authors declare that the research was conducted in the absence of any commercial or financial relationships that could be construed as a potential conflict of interest.

## Abbreviations

17OHP, 17-hydroxyprogesterone; 21OHD, 21-hydroxylase deficiency; BMI, Body mass index; CAH, Congenital adrenal hyperplasia; DSD, Disorders/differences in sex development; GBB-24, Giessen Subjective Complaints List; HADS, Hospital Anxiety and Depression Scale; IQR, Interquartile Range; SF-36, Short form 36; UK, United Kingdom; QoL, Quality of life; WHOQOL-BREF, World health organization quality of life questionnaire, short version.
