# Supplementary material for: Quality of Life in Men With Congenital Adrenal Hyperplasia Due to 21-Hydroxylase Deficiency
Source: Front Endocrinol (Lausanne). 2021 Mar 19;12:626646. doi: 10.3389/fendo.2021.626646 (PMC8018222; doi:10.3389/fendo.2021.626646)
Supplement: Supplementary file 1 [file DataSheet_1.docx]

**Supplement A: QoL domain scores - subgroups based on age and educational level in men with CAH.**

|  | Age | | p-value | | Educational level | | | p-value* | | |
| --- | --- | --- | --- | --- | --- | --- | --- | --- | --- | --- |
|  | < 30  (n=56) | ≥ 30  (n=53) | |  | Low  (n=14) | Medium  (n=59) | High  (n=25) | 1 | 2 |  |
| Physical health | 78.6  (71.4 – 91.1) | 75.0  (62.5 – 85.7) | | 0.17 | 73.2  (69.6 – 75.9) | 82.1  (67.9 – 92.9) | 71.4  (53.6 – 85.7) | 0.06 | 0.05 |  |
| Psychological health | 79.2  (70.8 – 95.8) | 75.0  (66.7 – 83.3) | | 0.09 | 79.2  (68.8 – 84.4) | 79.2  (70.8 – 87.5) | 70.8  (60.4 – 83.3) | 0.51 | 0.05 |  |
| Social relationships | 75.0  (60.4 – 91.7) | 66.7  (50.0 – 83.3) | | **0.03** | 75.0  (39.6 – 77.1) | 75.0  (58.3 – 91.7) | 66.7  (54.2 – 83.3) | 0.21 | 0.24 |  |
| Environment | 81.3  (71.9 – 90.6) | 78.1  (70.3 – 89.1) | | 0.44 | 78.1  (74.2 – 85.2) | 81.3  (71.9 – 93.8) | 81.3  (71.9 – 89.1) | 0.84 | 0.62 |  |

Median WHOQOL-BREF domain scores and interquartile range (Q1-Q3) for the subgroups based on age and education level were calculated. The Mann-Whitney-U test was used to compare groups. * p-value 1: low versus medium, p-value 2: medium versus high. **Bold**, p<0.05.


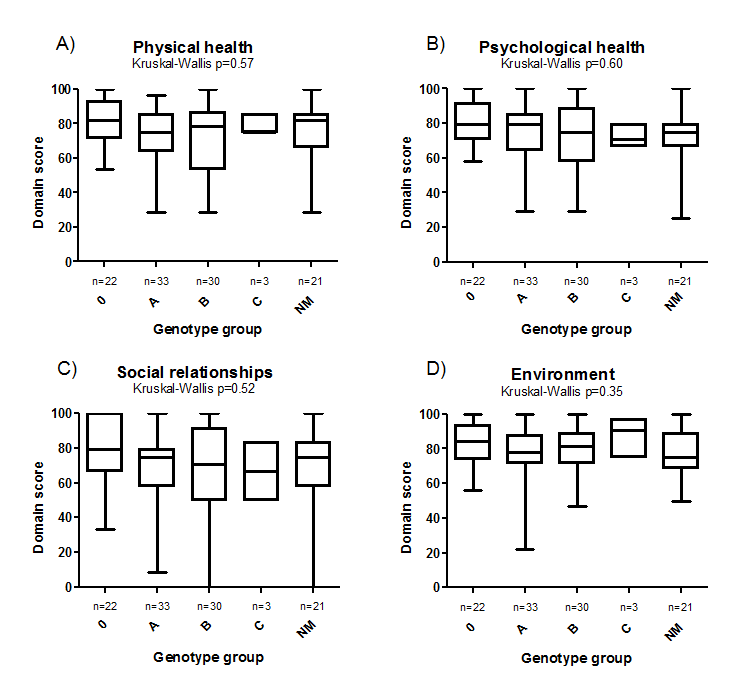


**Supplement B: Quality of Life domain scores among genotypes.** Patients with 21-hydroxylase deficiency were classified into genotype groups 0, A, B, or C.(15) Genotype C was excluded from comparative analysis due to low patient numbers (n=3). WHOQOL-BREF scores in different CAH genotype groups were calculated for four different domains: A) physical health, B) psychological health, C) social relationships, and D) environment. WHOQOL-BREF scores were converted to a 0-100 scale where higher scores reflect better QoL. Boxes represent median and 25th – 75th percentiles, while whiskers show minimum – maximum domain scores. Differences among the groups were assessed using the Kruskal-Wallis test. Abbreviations: NM, no mutation reported.
